# Rapamycin not dietary restriction improves resilience against pathogens: a meta-analysis

**DOI:** 10.1007/s11357-022-00691-4

**Published:** 2022-11-18

**Authors:** Eleanor J. Phillips, Mirre J. P. Simons

**Affiliations:** School of Biosciences, University of Sheffield, Western Bank, Sheffield, S10 2TN UK

**Keywords:** Diet, Rapamycin, Restriction, Aging, Immunosenescence, Infection

## Abstract

Dietary restriction (DR) and rapamycin both increase lifespan across a number of taxa. Despite this positive effect on lifespan and other aspects of health, reductions in some physiological functions have been reported for DR, and rapamycin has been used as an immunosuppressant. Perhaps surprisingly, both interventions have been suggested to improve immune function and delay immunosenescence. The immune system is complex and consists of many components. Therefore, arguably, the most holistic measurement of immune function is survival from an acute pathogenic infection. We reanalysed published post-infection short-term survival data of mice (*n* = 1223 from 23 studies comprising 46 effect sizes involving DR (*n* = 17) and rapamycin treatment (*n* = 29) and analysed these results using meta-analysis. Rapamycin treatment significantly increased post infection survival rate (lnHR =  − 0.72; CI =  − 1.17, -0.28; *p* = 0.0015). In contrast, DR reduced post-infection survival (lnHR = 0.80; CI = 0.08, 1.52; *p* = 0.03). Importantly, the overall effect size of rapamycin treatment was significantly lower (*p* < 0.001) than the estimate from DR studies, suggesting opposite effects on immune function. Our results show that immunomodulation caused by rapamycin treatment is beneficial to the survival from acute infection. For DR, our results are based on a smaller number of studies, but do warrant caution as they indicate possible immune costs of DR. Our quantitative synthesis suggests that the geroprotective effects of rapamycin extend to the immune system and warrants further clinical trials of rapamycin to boost immunity in humans.

**Supplementary Information:**

The online version contains supplementary material available at 10.1007/s11357-022-00691-4.

## Introduction 

Ageing is the progressive decline of function and increased risk of death. Many phenotypes are associated with ageing [1], including declining immune function [2, 3]. Immunosenescence leads to the dysfunction of immune cells affecting both innate and adaptive immunity [4–7] and to higher levels of inflammation [8]. Ageing therefore reduces our ability to mount an effective immune response, leaving us more susceptible to infection [3, 9]. More broadly immunosenescence is thought to underlie several pathologies that appear during ageing, including cancer [10], autoimmune disease [5] and ineffective clearance and accumulation of senescent cells [7, 11]. Immunosenescence thus provides an attractive explanation and potential therapeutic avenue for ageing.

Established treatments that extend lifespan in model organisms, most notably dietary restriction (DR) [12, 13] and mTOR suppression [14, 15], might do so because they mitigate immunosenescence. The pro-longevity mechanisms of DR have been hypothesised to include mTOR suppression [16, 17], but direct evidence for this hypothesis is scarce [14, 18–20]. Whether DR and mTOR suppression promote a healthier immune system and whether they do so through shared mechanisms is currently unclear. There are reports of beneficial effects of both of these pro-longevity interventions on immune function, yet there is also evidence to the contrary [21–23]. In addition, rapamycin (inhibiting mTOR) has been used as an immunosuppressant [23], and a loss of immune defence is a hypothesised cost of DR [24].

When measurements of the composition of the immune system are taken as proxies for immune health, extrapolation to overall organismal health is difficult. An additional complication is that such proxies are often studied under controlled, pathogen-free conditions [25, 26]. In comparison, acute survival to pathogens has received less attention but provides a strong experimental and potentially translational paradigm to study the effects of DR and rapamycin. Pathogen infection is a pervasive problem that intensifies with age [3, 27]. Treatments that enhance the effectiveness of the immune system to overcome infection are thus highly relevant. Conversely, should pro-longevity treatments simultaneously reduce the capacity to fight-off infection, the beneficial impact of DR and rapamycin on healthspan could be negated by reduced survival following naturally occurring infections [15]. We conducted a meta-analysis on studies in mice and found that survival after pathogen exposure was reduced by DR but improved with rapamycin.

## Results

**Fig. 1:**
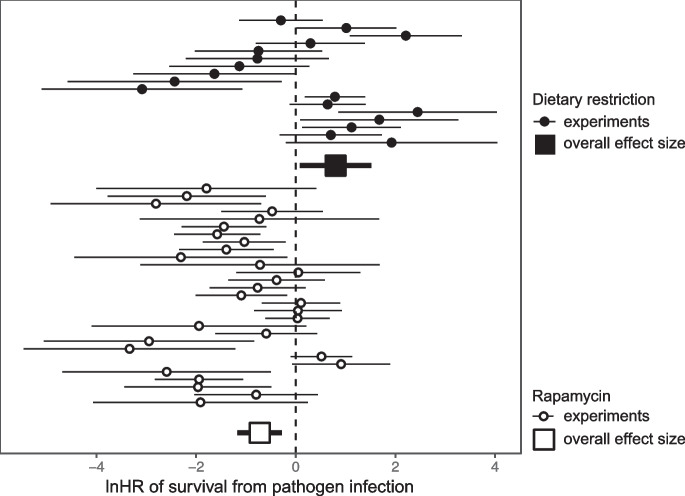
Forest plot of log hazard ratio estimates (circles) for DR and rapamycin post-infection survival curve pairings (*n* = 46) from Cox proportional hazard models. Squares indicate overall effect sizes as determined using meta-analysis controlling for interdependence of study and shared controls. Whiskers indicate 95% CIs

DR had a significant negative effect on survival following pathogen exposure (Fig. 1, lnHR = 0.80; CI = 0.08, 1.52; *p* = 0.03). There was a large proportion of relative heterogeneity (*I*^2^ = 0.68; Q-test df = 16, *p* < 0.01). The small sample size of (seven) studies and variation in the recorded moderators were too small to perform any meaningful moderator analysis. This together with heterogeneity between studies and interdependency of effect sizes from the same study and using the same controls reduces the overall confidence in this result. It is unlikely however that variation between studies was due to mouse genotype or degree of DR, as all studies used the common inbred mouse strain, C57BL/6, and DR of 40% (Table S1). However, the only study to find a significant positive effect of DR [28] used a parasitic model of infection and was the only study to use females. No publication bias was detected using a rank correlation (Kendall’s *τ*_*b*_ =  − 0.25; *p* = 0.18, Figure S3).

Rapamycin treatment improved survival of mice exposed to pathogens (lnHR =  − 0.72; CI =  − 1.17, − 0.28; *p* = 0.0015). Strikingly, when both interventions were analysed together, with treatment type as moderator, rapamycin-treated mice had significantly better survival than those treated with DR (estimate =  − 1.50; CI = − 2.33, − 0.68; *p* < 0.001). There was large relative heterogeneity (*I*^2^ = 0.67; Q = 84, df = 28, *p* < 0.01). To perhaps explain some of this heterogeneity, we tested a number of possible moderators. We found no significant contribution from mouse genotype (*Q*_*M*_ = 2.78, *df* = 4, *p* = 0.60; or when testing BL6 against other: *Q*_*M*_ = 0.63, df = 1; *p* = 0.43), inoculation method (*Q*_*M*_ = 0.76, *df* = 2; *p* = 0.69) or pathogen type (*Q*_*M*_ = 1.25, *df* = 3, *p* = 0.74). The effect of sex could not be evaluated as information was not provided or was female (see Table S2). There was a trend that secondary infection (*Q*_*M*_ = 3.49, *df* = 1, *p* = 0.06) showed a stronger effect of rapamycin (− 0.81; CI =  − 1.65, 0.04). A rank test of funnel plot asymmetry revealed no evidence for publication bias (Kendall’s *τ*_*b*_ = 0.23; *p* = 0.09; Figure S4).

## Discussion

Through meta-analysis, we found that rapamycin treatment but not DR significantly increased survival of mice exposed to pathogens. The pooled results of the limited number of studies suggest that DR does not improve immunity to infection and could even worsen the response. Studies on the impacts of rapamycin on infected mice have been inconclusive when comparing individual studies [29, 30]. Contrary to DR, however, our meta-analysis revealed that rapamycin protected against pathogenic infection. This disparity between DR and rapamycin supports previous suggestions, that these two anti-ageing treatments operate though largely distinct mechanisms [14, 19, 20, 31].

A common interpretation is that DR benefits immune function by keeping it ‘younger for longer’ [32, 33]. For instance, by protecting T-lymphocytes from oxidative damage [34], altering specific lymphocyte populations [35] and delaying thymic maturation [36]. However, our meta-analysis suggests that this ‘youthful’ immune system does not translate into a more potent response to pathogens. Perhaps aspects of innate immunity are compromised under DR. A reduced level of IL-6 [37] and reduced number, and cytotoxicity, of NK cells [38] under DR were associated with reduced survival of mice upon infection. While DR decreases effectiveness of NK cell-based immunity, arguably regulated by leptin [38–40], this could also prevent a hyperimmune response-enhancing survival. Similarly, a reduction in leptin production under DR was shown to be responsible for enhanced survival from cerebral malaria, and these effects were mediated through reduced mTORC1 activity in T cells [28].

Several mechanisms could explain why rapamycin increases resilience against pathogen infection. Immunosuppressive properties of rapamycin could prevent the activation of an overzealous immune response [29, 41]. A more effective immune response could stem from elevated numbers of T regulatory (Treg) cells seen after rapamycin treatment [29, 42]. Treg cells cause immune suppression to maintain homeostasis, for example reducing cytokine production which in turn ameliorates tissue damage [43]. Rapamycin may also improve immune memory [44–46], possibly fitting with the trend that secondary infections showed a stronger response to treatment. Rapamycin’s ability to reduce the debilitating effects of ageing on a systemic level could directly or indirectly benefit the immune system [47]. It remains to be determined to what degree the life-extending effects of rapamycin are due to its modulation of the immune system. Although, lifespan extension by rapamycin in mice lacking T and B lymphocytes (RAG2^−/−^) without a rescue from an immune challenge [48] suggests immunomodulation is not exclusively responsible for rapamycin’s anti-ageing effects. Outside the protected lab environment, however, infection and repeated exposure to pathogens could be strongly determinative of healthy ageing and lifespan. In this context, rapamycin has a strong immediate potential to benefit humans [47].

For the studies included in our meta-analysis, the duration and timing of treatment and age at pathogen exposure was so heterogeneous that we were unable to assess it (Table S2). Notably, in one study, short-term rapamycin treatment was more successful in improving post-infection survival than long-term treatment [49]. When comparing rapamycin to DR treatment, we note that the majority of the DR studies initiated treatment well in advance of infection, whereas treatment with rapamycin was more brief. In fact, the one study that started DR on the day of infection was also the only study to find a significant benefit to survival [28]. Timing and scheduling of rapamycin treatment can have unpredictable effects and could depend on age. Transient rapamycin treatment [50] and mTor knockdown [51] in early adult life extend lifespan in flies. Similarly, rapamycin during development [52] and a short bout of treatment at middle age [53] extend lifespan in mice. Determining which rapamycin schedule is most beneficial to the ageing human will be key. It is encouraging however that short-term rapamycin treatment in model organisms has benefits on both lifespan and on immune responses to pathogens, as we determined here through meta-analysis, paving the way for future human studies.

## Methods

### Literature research

Scopus and Google scholar were the two primary databases used to collect results for search terms relating to both DR and rapamycin. Additional sources were also found by searching the reference sections of salient papers (denoted as ‘Other Sources’ in the PRISMA report — Figure S1). As part of standard meta-analytic protocol [54], the PICO (Population, Intervention, Comparison, Outcome) framework was used to establish the specific research questions of the meta-analysis for both rapamycin (how rapamycin impacts the immune response of non-mutant mice compared to mice treated with placebo vehicle injection) and DR treatment (how DR impacts the immune response of non-mutant mice compared to mice fed ad libitum). From our initial literature research, we established that post infection survival is a common and relevant metric used. Although DR and rapamycin experiments have been conducted on species from a range of taxa, the most extensively studied and well-controlled subject group were laboratory mice. Given this, we focussed the meta-analysis on studies on mice that measured short-term survival following pathogen exposure.

### Inclusion criteria

General inclusion criteria are as follows: 

1. The experiment contained a control group and a group under DR or treated with rapamycin
2. The study included survival data in the form of a Kaplan–Meier plot, or provided original/raw survival data
3. Studies that used mouse strains that were selected or genetically modified in a way that would prompt an abnormal response were excluded. For instance, p53-deficient mice were excluded as they exhibit accelerated immune ageing [55].
4. There were no restrictions on the age or sex, but this information was collected for potential use in moderator analysis.
5. Survival data from the experiment could be in response to primary pathogen exposure or secondary exposure to the same or similar pathogen, for instance, in a study by Keating and colleagues [45].
6. The studies chosen were restricted to those which used microparasites as the pathogen for their immune challenge.
7. There were no restrictions on the date papers were published.
8. Studies with insufficient or unclear data were excluded, e.g. studies that did not include sample size or only survival data as an overall percentage rather than a Kaplan–Meier plot [56]. One study such, by Huang and colleagues [30], was due to a culmination of insufficient detail (rapamycin dose and mouse sex were not stated), a lack of independent controls and small sample size.

Treatment-specific inclusion criteria are as follows:

For DR experiments:

1. Restrict overall food intake as opposed to restricting a specific macro or micro-nutrients.
2. There was no limit on duration of DR prior to infection.
3. Studies with DR conditions of 40–60% ad libitum to represent moderate restriction.

For rapamycin experiments:

1. The experiment could use rapamycin at any dosage but not in conjunction with another drug.
2. There was also no restriction on duration of rapamycin treatment, but this information was recorded.

### Search methodology

The following key terms were entered into the chosen databases; the searches were modified to fit the format of an advanced search in each database. Scopus: (1) (“Dietary Restriction” OR “Undernutrition”) AND ((infection OR influenza)) AND (mice) AND NOT (review) returned 64 hits. (2) “Rapamycin” AND (infection OR influenza) AND (mice) AND NOT (review) returned 853 hits. Google Scholar: (1) (Dietary Restriction OR DR) AND (immune challenge OR infection) AND (mice OR Mouse) returned ~ 68,100 hits. (2) [Dietary Restriction] AND (infection OR immune response) AND [mice] AND “research paper” returned ~ 162,000 hits. (3) “Dietary Restriction” AND (infection OR influenza) AND [mice] AND review returned ~ 603 hits. Note, alternative names for/forms of rapamycin were also queried but these did not return any additional studies. Papers were assessed and selected manually following our inclusion and exclusion criteria and subsequently using the PRISMA guide (Figure S1). All literature searches were conducted by EP. A secondary non-structured search was conducted by MJPS as this can yield additional suitable literature. Later cross-referencing with the structured search yielded five additional suitable studies for the meta-analysis (Figure S1).

### Data extraction and re-analysis

Raw survival times were extracted using image analysis of published Kaplan–Meier survival curves. These analyses were performed using the WebPlotDigitizer analysis software. This software uses labelled axes from the published survival curve to then measure the location of points on each survival curve [14, 57]. The extracted data was re-analysed using Cox proportional hazards to assess the relationship between post infection survival probability and DR or rapamycin treatment (R package: survival; function: coxph) [58]. Individuals still alive at follow up were right-hand censored. No individuals were censored in these studies during the experiment. The effect size estimates and Kaplan–Meier survival curves generated from this analysis were compared to those in the original publications to confirm that data had been extracted accurately, and the direction of the effect corresponded to those reported in the original published work. We extracted pathogen type, infection method, sex and mouse genotype to be used in possible moderator analysis (Table S2). To include as many pertinent studies as possible, a range of pathogens were included, and pathogen type was extracted as a moderator. Longevity induced by rapamycin treatment has been shown to be differentially affected by sex, with greater lifespan increase in female mice than male mice at a variety of doses [19]. Genotype has also been shown to impact lifespan of mice treated with both DR [59] and rapamycin [57]. Additionally, there is evidence that the most common mouse models used in relevant studies, BALB/c and C57BL/6, exhibit distinctive immune responses when exposed to bacterial infection [60].

### Meta-analysis

Effect sizes, expressed as log hazard ratios from each study, were then analysed using a random-effects multilevel meta-analysis model (R package: metafor; function: rma.mv) [61]. Standard errors from the Cox proportional hazard models provided the weighting of each effect size in the analysis (the inverse of s.e. squared). As several effect sizes used the same control group, we accounted for this shared variance by including a covariance matrix [14] calculated using ‘vcalc’ in metafor, using a correlation of 0.5 between effect sizes of shared controls. Multilevel meta-analysis allows the inclusion of random effects and we included study as a random intercept for the multiple experiments from the same study. Where possible, post hoc subgroup analysis was performed to assess potential variables that may have contributed to heterogeneity. We only performed moderator analysis if the moderator could be objectively coded as a continuous variable or a factor with enough replication within levels to be tested. We indicate in the text where this was not possible due to heterogeneity in reporting or low number of replications. Relative heterogeneity was assessed using a multilevel version of *I*^2^ [62], and we also report *Q* tests. Publication bias within the meta-analysis was assessed visually using funnel plots (Figures S3 and S4) and statistically using a rank correlation test for funnel asymmetry using Kendall rank correlations.

## Supplementary Information

Below is the link to the electronic supplementary material.

Supplementary file1 (DOCX 497 KB)

## References

[1] López-Otín C, Blasco MA, Partridge L, Serrano M, Kroemer G (2013). The hallmarks of aging. Cell.

[2] Chung HY, Kim HJ, Kim KW, Choi JS, Yu BP (2002). Molecular inflammation hypothesis of aging based on the anti-aging mechanism of calorie restriction. Microsc Res Tech.

[3] Gavazzi G, Krause K-H (2002). Ageing and infection. Lancet Infect Dis.

[4] Nikolich-Žugich J, Messaoudi I (2005). Mice and flies and monkeys too: caloric restriction rejuvenates the aging immune system of non-human primates. Exp Gerontol, Metabolism, Aging and Longevity.

[5] Ritz BW, Gardner EM (2006). Malnutrition and energy restriction differentially affect viral immunity. J Nutr.

[6] Shaw AC, Goldstein DR, Montgomery RR (2013). Age-dependent dysregulation of innate immunity. Nat Rev Immunol.

[7] Yousefzadeh MJ, Flores RR, Zhu Y, Schmiechen ZC, Brooks RW, Trussoni CE, Cui Y, Angelini L, Lee K-A, McGowan SJ, Burrack AL, Wang D, Dong Q, Lu A, Sano T, O’Kelly RD, McGuckian CA, Kato JI, Bank MP, Wade EA, Pillai SPS, Klug J, Ladiges WC, Burd CE, Lewis SE, LaRusso NF, Vo NV, Wang Y, Kelley EE, Huard J, Stromnes IM, Robbins PD, Niedernhofer LJ (2021). An aged immune system drives senescence and ageing of solid organs. Nature.

[8] Baylis D, Bartlett DB, Patel HP, Roberts HC (2013). Understanding how we age: insights into inflammaging. Longev Heal.

[9] Aw D, Silva AB, Palmer DB (2007). Immunosenescence: emerging challenges for an ageing population. Immunology.

[10] Foster AD, Sivarapatna A, Gress RE (2011). The aging immune system and its relationship with cancer. Aging Health.

[11] Goronzy JJ, Weyand CM (2019). Mechanisms underlying T cell ageing. Nat Rev Immunol.

[12] Fontana L, Partridge L, Longo VD (2010). Extending healthy life span—from yeast to humans. Science.

[13] Katewa SD, Kapahi P (2010). Dietary restriction and aging, 2009. Aging Cell.

[14] Garratt M, Nakagawa S, Simons MJP (2016). Comparative idiosyncrasies in life extension by reduced mTOR signalling and its distinctiveness from dietary restriction. Aging Cell.

[15] Johnson SC, Rabinovitch PS, Kaeberlein M (2013). mTOR is a key modulator of ageing and age-related disease. Nature.

[16] Cox LS, Mattison JA (2009). Increasing longevity through caloric restriction or rapamycin feeding in mammals: common mechanisms for common outcomes?. Aging Cell.

[17] Green CL, Lamming DW, Fontana L (2022). Molecular mechanisms of dietary restriction promoting health and longevity. Nat Rev Mol Cell Biol.

[18] Bjedov I, Toivonen JM, Kerr F, Slack C, Jacobson J, Foley A, Partridge L (2010). Mechanisms of life span extension by rapamycin in the fruit fly Drosophila melanogaster. Cell Metab.

[19] Miller RA, Harrison DE, Astle CM, Fernandez E, Flurkey K, Han M, Javors MA, Li X, Nadon NL, Nelson JF, Pletcher S, Salmon AB, Sharp ZD, Van Roekel S, Winkleman L, Strong R (2014). Rapamycin-mediated lifespan increase in mice is dose and sex dependent and metabolically distinct from dietary restriction. Aging Cell.

[20] Unnikrishnan A, Kurup K, Salmon AB, Richardson A (2020). Is rapamycin a dietary restriction mimetic?. J Gerontol Ser A.

[21] Jolly CA (2004). Dietary restriction and immune function. J Nutr.

[22] Mannick JB, Del Giudice G, Lattanzi M, Valiante NM, Praestgaard J, Huang B, Lonetto MA, Maecker HT, Kovarik J, Carson S, Glass DJ, Klickstein LB (2014). mTOR inhibition improves immune function in the elderly. Sci Transl Med.

[23] Saunders RN, Metcalfe MS, Nicholson ML (2001). Rapamycin in transplantation: a review of the evidence. Kidney Int.

[24] Speakman JR, Mitchell SE (2011). Caloric restriction. Mol Aspects Med, Caloric Restriction.

[25] Camell CD, Yousefzadeh MJ, Zhu Y, Prata LGPL, Huggins MA, Pierson M, Zhang L, O’Kelly RD, Pirtskhalava T, Xun P, Ejima K, Xue A, Tripathi U, Espindola-Netto JM, Giorgadze N, Atkinson EJ, Inman CL, Johnson KO, Cholensky SH, Carlson TW, LeBrasseur NK, Khosla S, O’Sullivan MG, Allison DB, Jameson SC, Meves A, Li M, Prakash YS, Chiarella SE, Hamilton SE, Tchkonia T, Niedernhofer LJ, Kirkland JL, Robbins PD (2021). Senolytics reduce coronavirus-related mortality in old mice. Science.

[26] Goldberg EL, Romero-Aleshire MJ, Renkema KR, Ventevogel MS, Chew WM, Uhrlaub JL, Smithey MJ, Limesand KH, Sempowski GD, Brooks HL, Nikolich-Žugich J (2015). Lifespan-extending caloric restriction or mTOR inhibition impair adaptive immunity of old mice by distinct mechanisms. Aging Cell.

[27] Castle SC (2000). Clinical relevance of age-related immune dysfunction. Clin Infect Dis.

[28] Mejia P, Treviño-Villarreal JH, Hine C, Harputlugil E, Lang S, Calay E, Rogers R, Wirth D, Duraisingh MT, Mitchell JR (2015). Dietary restriction protects against experimental cerebral malaria via leptin modulation and T-cell mTORC1 suppression. Nat Commun.

[29] Canivet C, Menasria R, Rhéaume C, Piret J, Boivin G (2015). Valacyclovir combined with artesunate or rapamycin improves the outcome of herpes simplex virus encephalitis in mice compared to antiviral therapy alone. Antiviral Res.

[30] Huang C-T, Hung C-Y, Chen T-C, Lin C-Y, Lin Y-C, Chang C-S, He Y-C, Huang Y-L, Dutta A (2017). Rapamycin adjuvant and exacerbation of severe influenza in an experimental mouse model. Sci Rep.

[31] Birkisdóttir MB, Jaarsma D, Brandt RMC, Barnhoorn S, van Vliet N, Imholz S, van Oostrom CT, Nagarajah B, Portilla Fernández E, Roks AJM, Elgersma Y, van Steeg H, Ferreira JA, Pennings JLA, Hoeijmakers JHJ, Vermeij WP, Dollé MET (2021). Unlike dietary restriction, rapamycin fails to extend lifespan and reduce transcription stress in progeroid DNA repair-deficient mice. Aging Cell.

[32] Messaoudi I, Fischer M, Warner J, Park B, Mattison J, Ingram DK, Totonchy T, Mori M, Nikolich-Žugich J (2008). Optimal window of caloric restriction onset limits its beneficial impact on T-cell senescence in primates. Aging Cell.

[33] Pae M, Meydani SN, Wu D (2011). The role of nutrition in enhancing immunity in aging. Aging Dis.

[34] González O, Tobia C, Ebersole J, Novak M (2012). Caloric restriction and chronic inflammatory diseases. Oral Dis.

[35] Abe T, Nakajima A, Satoh N, Ohkoshi M, Sakuragi S, Koizumi A (2001). Suppression of experimental autoimmune uveoretinitis by dietary calorie restriction. Jpn J Ophthalmol.

[36] Chacón F, Cano P, Lopez-Varela S, Jiménez V, Marcos A, Esquifino AI (2002). Chronobiological features of the immune system. Effect of calorie restriction. Eur J Clin Nutr.

[37] Sun D, Muthukumar AR, Lawrence RA, Fernandes G. Effects of calorie restriction on polymicrobial peritonitis induced by cecum ligation and puncture in young C57BL/6 mice. Clin Diagn Lab Immunol 2001;8:1003–11. 10.1128/CDLI.8.5.1003-1011.2001 10.1128/CDLI.8.5.1003-1011.2001 PMC96186 11527818

[38] Clinthorne JF, Adams DJ, Fenton JI, Ritz BW, Gardner EM (2010). Short-term re-feeding of previously energy-restricted C57BL/6 male mice restores body weight and body fat and attenuates the decline in natural killer cell function after primary influenza infection. J Nutr.

[39] Clinthorne JF, Beli E, Duriancik DM, Gardner EM (2013). NK cell maturation and function in C57BL/6 mice are altered by caloric restriction. J Immunol.

[40] Naylor C, Petri WA (2016). Leptin regulation of immune responses. Trends Mol Med.

[41] Kalil AC, Thomas PG (2019). Influenza virus-related critical illness: pathophysiology and epidemiology. Crit Care.

[42] Goldberg EL, Smithey MJ, Lutes LK, Uhrlaub JL, Nikolich-Žugich J (2014). Immune memory–boosting dose of rapamycin impairs macrophage vesicle acidification and curtails glycolysis in effector CD8 cells, impairing defense against acute infections. J Immunol.

[43] Liu Q, Zhou Y, Yang Z (2016). The cytokine storm of severe influenza and development of immunomodulatory therapy. Cell Mol Immunol.

[44] Chen C, Liu Yu, Liu Yang, Zheng P. mTOR regulation and therapeutic rejuvenation of aging hematopoietic stem cells. Sci Signal 2009; 2. 10.1126/scisignal.2000559 10.1126/scisignal.2000559 PMC4020596 19934433

[45] Keating R, Hertz T, Wehenkel M, Harris TL, Edwards BA, McClaren JL, Brown SA, Surman S, Wilson ZS, Bradley P, Hurwitz J, Chi H, Doherty PC, Thomas PG, McGargill MA (2013). The kinase mTOR modulates the antibody response to provide cross-protective immunity to lethal infection with influenza virus. Nat Immunol.

[46] Liepkalns JS, Pandey A, Hofstetter AR, Kumar A, Jones EN, Cao W, Liu F, Levine MZ, Sambhara S, Gangappa S (2016). Rapamycin does not impede survival or induction of antibody responses to primary and heterosubtypic influenza infections in mice. Viral Immunol.

[47] Bischof E, Siow RC, Zhavoronkov A, Kaeberlein M (2021). The potential of rapalogs to enhance resilience against SARS-CoV-2 infection and reduce the severity of COVID-19. Lancet Healthy Longev.

[48] Hurez V, Dao V, Liu A, Pandeswara S, Gelfond J, Sun L, Bergman M, Orihuela CJ, Galvan V, Padrón Á, Drerup J, Liu Y, Hasty P, Sharp ZD, Curiel TJ (2015). Chronic mTOR inhibition in mice with rapamycin alters T, B, myeloid, and innate lymphoid cells and gut flora and prolongs life of immune-deficient mice. Aging Cell.

[49] Hinojosa CA, Mgbemena V, Van Roekel S, Austad SN, Miller RA, Bose S, Orihuela CJ (2012). Enteric-delivered rapamycin enhances resistance of aged mice to pneumococcal pneumonia through reduced cellular senescence. Exp Gerontol.

[50] Juricic P, Lu Y-X, Leech T, Drews LF, Paulitz J, Lu J, Nespital T, Azami S, Regan JC, Funk E, Fröhlich J, Grönke S, Partridge L. Long-lasting geroprotection from brief rapamycin treatment in early adulthood by persistently increased intestinal autophagy. Nat Aging 2022; 1–13. 10.1038/s43587-022-00278-w 10.1038/s43587-022-00278-w PMC10154223 37118497

[51] Simons MJP, Hartshorne L, Trooster S, Thomson J, Tatar M. 2019. Age-dependent effects of reduced mTor signalling on life expectancy through distinct physiology. preprint bioRxiv. 10.1101/719096

[52] Shindyapina AV, Cho Y, Kaya A, Tyshkovskiy A, Castro JP, Gordevicius J, Poganik JR, Horvath S, Peshkin L, Gladyshev VN (2022). Preprint bioRxiv. Rapamycin treatment during development extends lifespan and healthspan.

[53] Bitto A, Ito TK, Pineda VV, LeTexier NJ, Huang HZ, Sutlief E, Tung H, Vizzini N, Chen B, Smith K, Meza D, Yajima M, Beyer RP, Kerr KF, Davis DJ, Gillespie CH, Snyder JM, Treuting PM, Kaeberlein M. Transient rapamycin treatment can increase lifespan and healthspan in middle-aged mice. eLife 2016;5:e16351. 10.7554/eLife.16351 10.7554/eLife.16351 PMC4996648 27549339

[54] Shamseer L, Moher D, Clarke M, Ghersi D, Liberati A, Petticrew M, Shekelle P, Stewart LA (2015). Preferred reporting items for systematic review and meta-analysis protocols (PRISMA-P) 2015: elaboration and explanation. BMJ.

[55] Ohkusu-Tsukada K, Tsukada T, Isobe K (1999). Accelerated development and aging of the immune system in p53-deficient mice. J Immunol Baltim Md.

[56] Davies WL, Smith SC, Pond WL, Rasmussen AF, Clark PF (1949). Effect of dietary restriction on susceptibility of mice to infection with Theiler’s GDVII virus. Proc Soc Exp Biol Med.

[57] Swindell WR (2017). Meta-analysis of 29 experiments evaluating the effects of rapamycin on life span in the laboratory mouse. J Gerontol Ser A.

[58] Therneau TM, Grambsch PM (2000) Modeling survival data: extending the cox model. Springer, New York

[59] Swindell WR (2012). Dietary restriction in rats and mice: a meta-analysis and review of the evidence for genotype-dependent effects on lifespan. Ageing Res Rev.

[60] Fornefett J, Krause J, Klose K, Fingas F, Hassert R, Eisenberg T, Schrödl W, Grunwald T, Müller U, Baums CG (2018). Comparative analysis of clinics, pathologies and immune responses in BALB/c and C57BL/6 mice infected with Streptobacillus moniliformis. Microbes Infect.

[61] Viechtbauer W (2010). Conducting meta-analyses in R with the metafor Package. J Stat Softw.

[62] Nakagawa S, Santos ESA (2012). Methodological issues and advances in biological meta-analysis. Evol Ecol.

