# Supplementary material for: Rapamycin not dietary restriction improves resilience against pathogens: a meta-analysis
Source: GeroScience. 2022 Nov 18;45(2):1263–70. doi: 10.1007/s11357-022-00691-4 (PMC9886774; doi:10.1007/s11357-022-00691-4)

**

Supplementary material**; Rapamycin not Dietary Restriction improves resilience against pathogens: a meta-analysis
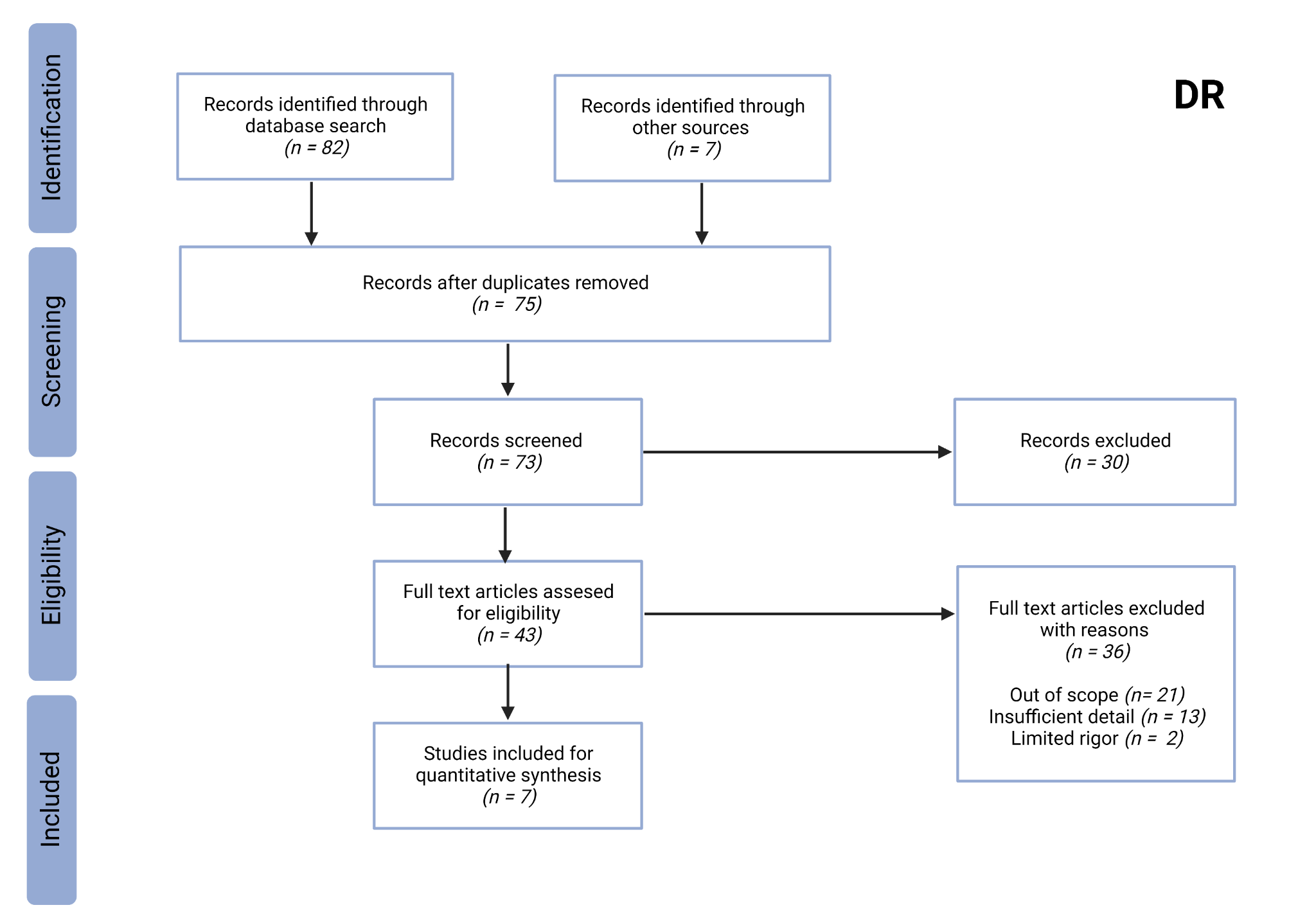


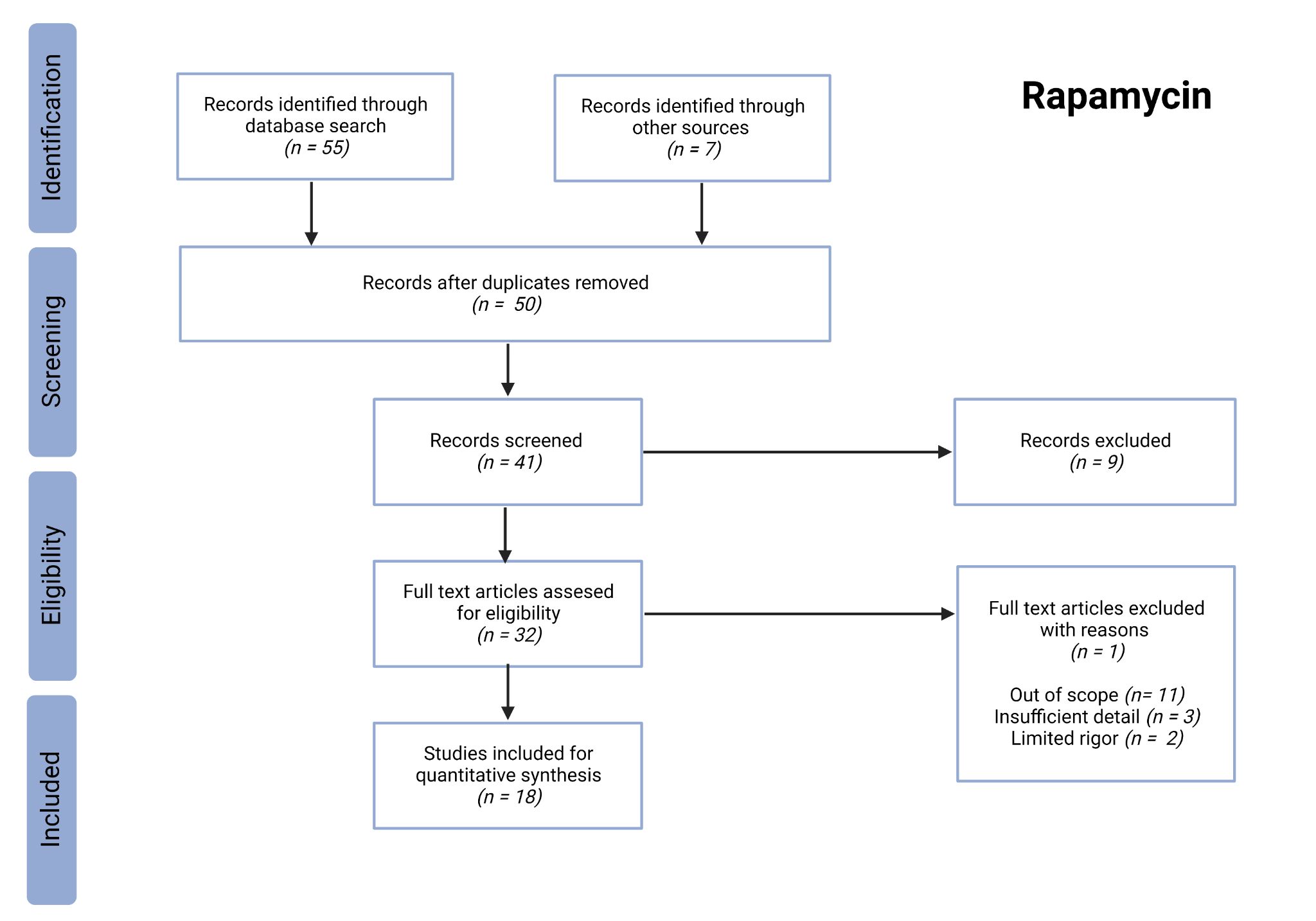


| ***Table S1****: Additional details of Dietary Restriction studies used in the meta-analysis, including effect sizes and details of DR treatments, infections and mouse populations used in each experiment.* | | | | | | | | | | |
| --- | --- | --- | --- | --- | --- | --- | --- | --- | --- | --- |
| Dataset | Effect Size  (ln HR) | |  | Details of Infection | | | | Details of Mouse Populations | | |
|  | Estimate | SE | Degree of Restriction (%) | Age at infection | Strength of Infection | Pathogen Type | Infection Method | Sample Size | Sex | Genotype |
| Clinthorne et al., 2010 | 1.92 | 1.08 | 40 | 6 months | 100 hgu | Viral (*Influenza,* H1N1 PR8) | Intranasal | 24 | Male | C57BL/6 |
| Gardner et al., 2005 (1) | 1.68 | 0.81 | 40 | 23 months | 0.1 hgu | Viral (Influenza, H1N1 PR8) | Intranasal | 21 | Male | C57BL/6 |
| Gardner et al., 2005 (2) | 0.70 | 0.52 | 40 | 23 months | 1 hgu | Viral (*Influenza,* H1N1 PR8) | Intranasal | 21 | Male | C57BL/6 |
| Gardner et al., 2005 (3) | 1.12 | 0.51 | 40 | 23 months | 10 hgu | Viral (Influenza, H1N1 PR8) | Intranasal | 21 | Male | C57BL/6 |
| Gardner et al., 2005 (4) | 2.45 | 0.81 | 40 | 23 months | 100 hgu | Viral (Influenza, H1N1 PR8) | Intranasal | 22 | Male | C57BL/6 |
| Goldberg et al., 2015 | 0.79 | 0.31 | 40 | 25 weeks | 1000 pfu | Viral (*Flavivirus,* West Nile Virus) | Injection | 60 | Male | C57BL/6 |
| Goldberg et al., 2015 | 0.64 | 0.39 | 40 | 25 weeks | 1000 pfu | Viral (*Flavivirus,* West Nile Virus) | Injection | 38 | Male | C57BL/6 |
| Mejia et al., 2015 (1) * | -1.63 | 0.83 | 40 | 8-10 weeks | 0.5 million RBCs | Parasitic (*Plasmodium. berghei*) | Injection | 52 | Female | C57BL/6J |
| Mejia et al., 2015 (2) | -1.13 | 0.71 | 40 | 8-10 weeks | 0.5 million RBCs | Parasitic (*Plasmodium. berghei*) | Injection | 39 | Female | C57BL/6J |
| Mejia et al., 2015 (3) | -0.75 | 0.65 | 40 | 8-10 weeks | 0.5 million RBCs | Parasitic (*Plasmodium. berghei*) | Injection | 16 | Female | C57BL/6J |
| Mejia et al., 2015 (4) | -2.43 | 1.10 | 40 | 8-10 weeks | 0.5 million RBCs | Parasitic (*Plasmodium. berghei*) | Injection | 16 | Female | C57BL/6J |
| Mejia et al., 2015 (5) | 0.25 | 0.56 | 40 | 8-10 weeks | 0.5 million RBCs | Parasitic (*Plasmodium. berghei*) | Injection | 16 | Female | C57BL/6J |
| Mejia et al., 2015 (6) | -3.08 | 1.03 | 40 | 8-10 weeks | 0.5 million RBCs | Parasitic (*Plasmodium. berghei*) | Injection | 16 | Female | C57BL/6J |
| Mejia et al., 2015 (7) | -0.77 | 0.73 | 50 | 8-10 weeks | 0.5 million RBCs | Parasitic (*Plasmodium. berghei*) | Injection | 16 | Female | C57BL/6J |
| Rao et al., 2017 | 1.51 | 0.53 | 40 | 6-8 weeks | 150 cfu | Bacterial (*Salmonella Typhimurium*) | Injection | 29 | Male | C57BL/6 |
| Ritz et al., 2008 | 1.01 | 0.51 | 40 | 6 months | 100 hgu | Viral (Influenza, H1N1 PR8) | Intranasal | 30 | Male | C57BL/6 |
| Trujillo-Ferrara et al., 2011 | -0.30 | 0.43 | 40 | 6 months | 10000 cfu | Bacterial (*Salmonella Typhimurium*) | Injection | 50 | NS | C57BL/6 |
| *Rows highlighted in red indicate experiments where DR had a significantly negative effect on post infection survival.* | | | | | | | | | | |
| *Rows highlighted in green indicate experiments where DR had a significantly positive effect on post infection survival.*  *NS = Not Stated*  ** Mejia and colleagues conducted the only DR study to test multiple start points for restriction at 7 days (1, 6, 7), 4 days (2) and 2 days (3) before infection, on the day of infection (4) and 2 days post infection (5). Only data from fig. d and fig. i were included.* | | | | | | | | | | |

| ***Table S2****: Additional details of rapamycin studies used in the meta-analysis, including effect sizes and details of DR treatments, infections and mouse populations used in each experiment.* | | | | | | | | | | | | |
| --- | --- | --- | --- | --- | --- | --- | --- | --- | --- | --- | --- | --- |
| Dataset | Effect Size  (ln HR) | | Details of Rapamycin Treatment | |  | Details of Infection | | | | Details of Mouse Population | | |
|  | Estimate | SE | Rapamycin Dose* | Frequency of Treatment | Age at Infection | Priming Infection | Pathogen Type | Infection Strength | Infection Method | Sample Size | Sex | Genotype |
| Bell et al., 2017 | -1.91 | 1.10 | 10 mg/kg daily | 1 hour before infection to 10 days post infection | 6-8 weeks | No | Viral (Rift Valley Fever Virus) | 150 pfu | Injection | 20 | Female | BALB/c |
| Bell et al., 2017 | -0.80 | 0.63 | 10 mg/kg daily | 1 hour before infection to 14 days post infection | 6-8 weeks | No | Viral (Rift Valley Fever Virus) | 1500 pfu | Injection | 20 | Female | BALB/c |
| Canivet et al., 2015 | -1.96 | 0.75 | 10 mg/kg daily | Days 4 to 13 post infection | 4-5 weeks | No | Viral (Herpes Simplex Virus) | 1.5 × 10^3^ pfu | Intranasal | 28 | Female | BALB/c |
| Canivet et al., 2015 | -1.94 | 0.45 | 10 mg/kg daily | Days 4 to 13 post infection | 4-5 weeks | No | Viral (Herpes Simplex Virus) | 1.5 × 10^3^ pfu | Intranasal | 45 | Female | BALB/c |
| Chen et al., 2009 | -2.59 | 1.07 | 4mg/kg every 2 days | 8 weeks before infection | 22-24 months | Yes | Viral (*Influenza* H1N1 PR8) | 400 hau | Intranasal | 24 | NS | C57BL/6 |
| Goldberg et al., 2014 | 0.91 | 0.50 | 75μg/kg daily | 2 days before infection to 7 days post infection | 16-18 months | No | Viral (*Flavivirus*, West Nile Virus) | 10^3^ pfu | Injection | 42 | NS | C57BL/6 |
| Goldberg et al., 2015 | 0.52 | 0.32 | 75μg/kg daily | 2 months before infection | 16-18 months | No | Viral (*Flavivirus*, West Nile Virus) | 400 hau | Intranasal | 57 | NS | C57BL/6 |
| Gordon et al., 2015 | -3.34 | 1.08 | 1mg/kg daily | 1 day post infection (until experiment end) | 7-10 weeks | No | Parasitic (*Plasmodium berghei)* | 1 × 10^6^ RBCs | Injection | 19 | Female | C57BL/6 |
| Gordon et al., 2015 | -2.95 | 1.08 | 1mg/kg daily | 4 days post infection (until experiment end) | 7-10 weeks | No | Parasitic (*Plasmodium berghei)* | 1 × 10^6^ RBCs | Injection | 18 | Female | C57BL/6 |
| Gordon et al., 2015 | -0.59 | 0.52 | 1mg/kg daily | 5 days post infection (until experiment end) | 7-10 weeks | No | Parasitic (*Plasmodium berghei)* | 1 × 10^6^ RBCs | Injection | 18 | Female | C57BL/6 |
| Gust et al., 2011 | -1.94 | 1.10 | 75μg/kg daily | 1 day before priming infection until secondary infection (28 days) | NS | Yes | Viral (*Influenza* H5N1) | 1 × 10^8^ EID_50_ | Injection | 20 | Female | C57BL/6 |
| Harrison et al., 2014 | 0.04 | 0.33 | NS | 6 weeks prior to infection and withdrawn before infection | 24.5 months | No | Bacterial (*Mycobacterium tuberculosis* (GP)) | NS | Inhalation | 50 | Female | HET3 |
| Heydarabadi et al., 2020 | 0.04 | 0.45 | 40μg/μl daily | NS | NS | No | Viral (*Rhabdoviridae*, RABV) | NS | Injection | 20 | NS | NMRI |
| High and Washburn, 1996 | 0.10 | 0.40 | 10mg/kg | 1 day before infection to 14 days post infection | NS | No | Fungal (*Aspergillus fumigatus*) | 7.5 × 10^6^ conidia | Injection | 40 | NS | CD-1 |
| Hinojosa et al., 2012 | -1.09 | 0.47 | 2.2mg/kg daily | 17 weeks before infection | 24 months | No | Bacterial (*Streptococcus. pneumoniae* (GP)) | 1 × 10^3^ cfu | Inhalation | 25 | Both | C57BL/6 |
| Hinojosa et al., 2012 | -0.77 | 0.49 | 2.2mg/kg daily | 86 weeks before infection | 24 months | No | Bacterial (*Streptococcus. pneumoniae* (GP)) | 1 × 10^3^ cfu | Inhalation | 29 | Both | C57BL/6 |
| Jai et al., 2018 | 0.05 | 0.64 | 600μg/kg on day 1 then 300μg/kg daily | 2 hours post infection (until experiment end | 6-8 weeks | No | Viral (*Influenza* H1N1 pdm09) | 10^2^ TCID_50_ | Intranasal | 16 | Female | BALB/c |
| Jai et al., 2018 | -0.38 | 0.49 | 600μg/kg on day 1 then 300μg/kg daily | 2 days post infection (until experiment end) | 6-8 weeks | No | Viral (*Influenza* H1N1 pdm09) | 10^2^ TCID_50_ | Intranasal | 32 | Female | BALB/c |
| Junkins et al., 2013 | -0.72 | 1.22 | 10mg/kg daily | 3 days before infection (until experiment end) | 8-10 weeks | No | Bacterial (*Pseudomonas aeruginosa* (GN)) | 10^9^ cfu | Intranasal | 30 | NS | C57BL/6 |
| Keating et al., 2013 | -2.31 | 1.09 | 75μg/kg daily | 1 day before primer infection | 8-10 weeks | No | Viral (*Influenza* A/HK/x31) | 1 × 10^8^ EID_50_ | Injection | 18 | Female | C57BL/6J |
| Keating et al., 2013 | -1.39 | 0.48 | 75μg/kg daily | 1 day before priming infection to 28 days post priming infection | 12-14 weeks | Yes | Viral (*Influenza* H5N1) | 4.5 × 10^5^ EID_50_ | Intranasal | 32 | Female | C57BL/6J |
| Keating et al., 2013 | -1.03 | 0.42 | 75μg/kg daily | 1 day before priming infection to 28 days post priming infection | 12-14 weeks | Yes | Viral (*Influenza* PR8) | 4.5 × 10^5^ EID_50_ | Intranasal | 36 | Female | C57BL/6J |
| Kim et al., 2020 | -1.58 | 0.44 | 150 μg | 3 and 5 hours after priming and 18 hours after secondary infection | 10 weeks | Yes | Fungal (*Candida albicans*) | 2 × 10^4^ cfu | Injection | 28 | Female | C57BL/6 |
| Kim et al., 2020 | -1.44 | 0.43 | 150 μg | 3 and 5 hours after priming and 18 hours after secondary infection | 10 weeks | Yes | Fungal (*Candida albicans*) | 2 × 10^4^ cfu | Injection | 28 | Female | C57BL/6 |
| Liepkalns et al., 2016 | -0.47 | 0.52 | 1.5 μg daily | 3 days before infection (until experiment end) | 6 weeks | No | Viral (*Influenza* H1N1 PR8) | 1.5 LD_50_ | Intranasal | 26 | NS | C57BL/6 |
| Liepkalns et al., 2016 | -0.73 | 1.22 | 12 μg daily | 3 days before infection (until experiment end) | 6 weeks | Yes | Viral (*Influenza* H1N1 PR8) | 1.5 LD_50_ | Intranasal | 20 | NS | C57BL/7 |
| Mejia et al., 2015 | -2.81 | 1.08 | 1 mg/kg | Days 1 to 3 post infection | 8-10 weeks | No | Parasitic (*Plasmodium berghei)* | 0.5 million RBCs | Injection | 20 | Female | C57BL/6 |
| Mejia et al., 2015 | -2.19 | 0.81 | 5 mg/kg | Days 1 to 3 post infection | 8-10 weeks | No | Parasitic (*Plasmodium berghei)* | 0.5 million RBCs | Injection | 20 | Female | C57BL/6 |
| Moraschi et al., 2021 | -1.79 | 1.13 | 0.075 mg/kg daily | 34 days starting at priming infection | 8 weeks | Yes | Parasitic (*Trypanosoma cruzi*) | 150 blood trypomastigotes | Injection | 14 | Both | C57BL/6 |
| *Rows highlighted in green indicate experiments where rapamycin had a significantly positive effect on post infection survival.* | | | | | | | | | | | | |
| *No experiment showed rapamycin to have a significantly negative effect on survival.* | | | | | | | | | | | | |
| *NS = Not Stated*  **Refers to body weight per mouse.* | | | | | | | | | | | | |
|  | | | | | | | | | | | | |



Supplement: Supplementary file 1 — Supplementary file1 (DOCX 497 KB) [file 11357_2022_691_MOESM1_ESM.docx]
